# Novel 4H-SiC Double-Trench MOSFETs with Integrated Schottky Barrier and MOS-Channel Diodes for Enhanced Breakdown Voltage and Switching Characteristics

**DOI:** 10.3390/nano15120946

**Published:** 2025-06-18

**Authors:** Peiran Wang, Chenglong Li, Chenkai Deng, Qinhan Yang, Shoucheng Xu, Xinyi Tang, Ziyang Wang, Wenchuan Tao, Nick Tao, Qing Wang, Hongyu Yu

**Affiliations:** 1 School of Integrated Circuit, Shenzhen Polytechnic University, Shenzhen 518055, China; 12333281@mail.sustech.edu.cn; 2 School of Microelectronics, Southern University of Science and Technology, Shenzhen 518055, China; 12011505@mail.sustech.edu.cn (C.L.); 12149033@mail.sustech.edu.cn (C.D.); 12440881@mail.sustech.edu.cn (Q.Y.); 12112715@mail.sustech.edu.cn (S.X.); 12232507@mail.sustech.edu.cn (X.T.); 12333347@mail.sustech.edu.cn (Z.W.); 12333343@mail.sustech.edu.cn (W.T.); 3 School of Electronic Information and Engineering, Harbin Institute of Technology, Harbin 150001, China; 4 Maxscend Microelectronics Co., Ltd., Wuxi 214072, China; nick.tao@maxscend.com; 5 Engineering Research Center of Integrated Circuits for Next-Generation Communications, Ministry of Education, Southern University of Science and Technology, Shenzhen 518055, China

**Keywords:** bipolar degradation, Schottky barrier diode (SBD), MOS-channel diode (MCD), SiC, double-trench MOSFET (DTMOS)

## Abstract

In this study, a novel silicon carbide (SiC) double-trench MOSFET (DT-MOS) combined Schottky barrier diode (SBD) and MOS-channel diode (MCD) is proposed and investigated using TCAD simulations. The integrated MCD helps inactivate the parasitic body diode when the device is utilized as a freewheeling diode, eliminating bipolar degradation. The adjustment of SBD position provides an alternative path for reverse conduction and mitigates the electric field distribution near the bottom source trench region. As a result of the Schottky contact adjustment, the reverse conduction characteristics are less influenced by the source oxide thickness, and the breakdown voltage (BV) is largely improved from 800 V to 1069 V. The gate-to-drain capacitance is much lower due to the removal of the bottom oxide, bringing an improvement to the turn-on switching rise time from 2.58 ns to 0.68 ns. These optimized performances indicate the proposed structure with both SBD and MCD has advantages in switching and breakdown characteristics.

## 1. Introduction

The silicon carbide (SiC) metal-oxide-semiconductor field-effect transistors (MOSFETs) are commonly recognized as promising devices for high-voltage, high-temperature, and high-frequency application scenarios [1,2,3]. SiC-based devices are more competitive compared with Si-based devices, as they are a new class of power electronics components that solve the intrinsic limitations of silicon technology [4]. SiC MOSFETs have shown impressive characteristics like wider bandgap, higher thermal conductivity, and better switching performance, bringing significant advantages in power applications to reduce power loss and decrease system scale [5,6,7]. SiC trench MOSFETs feature lower specific ON-resistance (*R*_on,sp_) by eliminating the junction field-effect transistor (JFET) region while bringing much more complicated manufacture processes [8]. Conventional SiC trench MOSFETs also have drawbacks like bipolar degradation and voltage drop due to the parasitic body diode [9]. These problems would make SiC MOSFETs not suitable for being driven as freewheeling diodes. Several methods to inactivate parasitic body diode and eliminate bipolar degradation have been introduced. A simple approach is to pair a SiC SBD externally, but it would increase the size of the system and integration cost [10,11].

Recently, the idea of integrating different kinds of diodes inside a device has been becoming increasingly popular. To suppress the bipolar degradation and the turn-on of the parasitic body diode, the integration of a Schottky barrier diode (SBD) or junction barrier Schottky diode (JBS) would solve the problems and improve the reverse performance [12,13,14,15,16,17,18]. Besides the SBD, a different kind of diode named the MOS-channel diode (MCD) is another feasible approach with which to eliminate the bipolar degradation and inactivate the parasitic body diode [19,20,21,22,23,24]. These methodologies of internal integration improve the freewheeling situation, while not benefiting the enhancement of breakdown voltage. The Schottky contact would be more vulnerable than the p-n diode structure when the device is under forward voltage, which would slightly decrease the breakdown voltage.

In this article, a SiC double-trench MOSFET embedded with both an MCD and SBD (MS-DTMOS) is proposed to further enhance the reverse performance and breakdown characteristics, and it is compared using Silvaco TCAD simulation [25] with a device with only an MCD (MCD-DTMOS). The integration of the MCD and SBD near the source trench region reduces the system cost of pairing an external SBD. While retaining the effect of inactivating the body diode and suppressing bipolar degradation, the pitch size is smaller than that integrating the two diodes separately. The introduction of the source SBD further enhances the switching performance and improves the breakdown characteristics, while bringing no extra volume. As a result of the Schottky contact adjustment, the reverse conduction characteristics are less influenced by the source oxide thickness, and the breakdown voltage (BV) is largely improved from 800 V to 1069 V. The gate-to-drain capacitance is much lower due to the removal of the bottom oxide, bringing an improvement to the turn-on switching rise time from 2.58 ns to 0.68 ns. These optimized performances indicate that the proposed structure with both an SBD and MCD has advantages in switching and breakdown characteristics.

## 2. Device Structure Design

**Figure 1:**
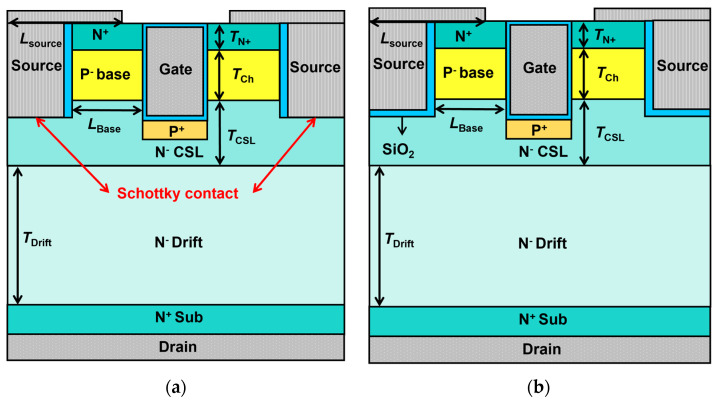
Schematic cross-sectional view of SiC (**a**) MS-DTMOS and (**b**) MCD-DTMOS.

The schematics of the proposed SiC MS-DTMOS and conventional MCD-DTMOS devices are shown in Figure 1. From bottom to top, both MOSFETs are constructed with an N+ substrate layer (N+ Sub), N drift layer, N current spreading layer (N-CSL), P base layer (P-base), and N+ layer. In both types of the SiC trench MOSFET, the MCD is integrated into the sidewall of the source trench region. The MOS-channel is formed by the source metal, source sidewall oxide, and the n-p-n structure. In the MS-DTMOS device, the bottom source oxide is removed to integrate the SBD. Thus, the SBD and MCD are both integrated into the SiC DTMOS near the source trench region. As the two diodes are integrated into one side of the device cell, the total device volume is reduced, resulting in a lower cost compared to the method of integrating two diodes separately. Table 1 lists the detailed parameters of the simulated devices.

When the device is working in reverse mode, the MCD or SBD would conduct before to the p-n diode near the gate trench region. The conduction of the p-n diode is suppressed. For the MS-DTMOS, the presence of the SBD provides an alternative current path concerning specific source oxide thickness (*t*_sox_), improving the reserve characteristics. However, the ability to control reverse current is different for the MCD affected by *t*_sox_, which would cause the phenomenon of dominance change between the SBD and MCD when the device is in reverse conduction mode.

## 3. Simulation Results and Discussion

### 3.1. Forward Output and Reverse Conduction Characteristics

**Figure 2:**
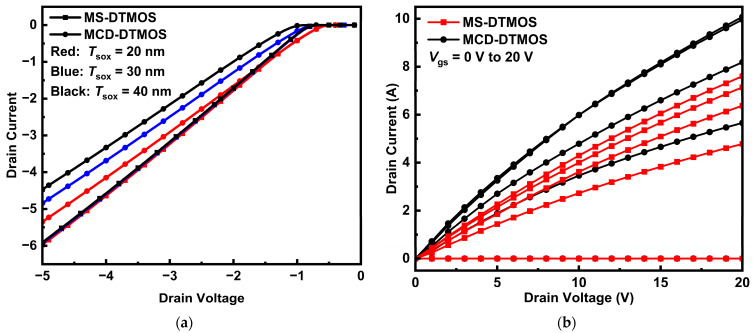
(**a**) Reverse conduction and (**b**) forward output characteristics of MS-DTMOS and MCD-DTMOS devices with different *t*_sox_ values.

The forward output and reverse conduction characteristics of the two devices at different values of gate voltage are shown in Figure 2. MS-DTMOS shows a larger reverse current than the MCD-DTMOS. The SBD structure inside the MS-DTMOS turns on and takes part in the reverse conduction. It provides an alternative path for reverse current, increasing the reverse current density. The MOS-channel diode turns on as the device switches to reverse mode as well. The capability to control the MCD is related to the source oxide thickness, and it becomes more apparent in the MS-DTMOS as the two diodes both exist. For MCD-DTMOS, the reverse conduction characteristic related to source oxide thickness is shown in Figure 2a. As the source oxide thickness increases, reverse current shifts to the negative side, which means the capability to control conduction is reduced. It becomes harder to be turned on when increasing the source oxide thickness. In Figure 2a, the reverse current characteristics of MS-DTMOS becomes similar when the source oxide thickness is above 20 nm. This is because the Schottky barrier diode maintains dominance when the source oxide is too thick for the MCD to conduct, while the SBD only requires a lower voltage to work.

**Figure 3:**
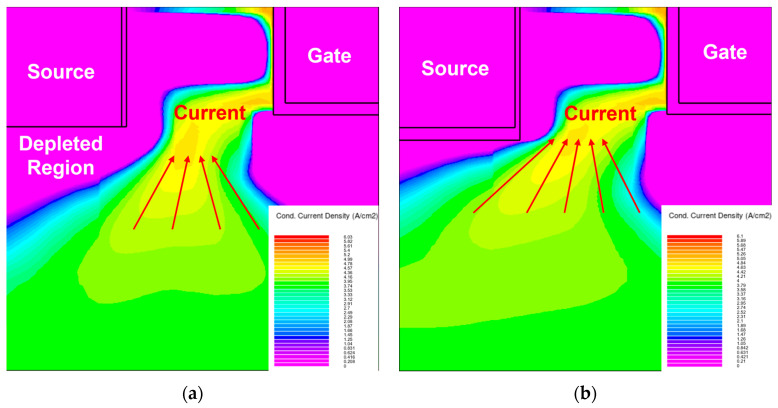
Current density distribution of (**a**) MS-DTMOS and (**b**) MCD-DTMOS when the devices are in output mode.

The forward output characteristics of MS-DTMOS and MCD-DTMOS are presented in Figure 2b. Due to the current spreading area of MS-DTMOS being smaller than the current spreading area of MCD-DTMOS, as shown in Figure 3, the forward output current of MS-DTMOS is slightly lower than that of MCD-DTMOS. The introduction of the Schottky contact brings a slight drop in current density, resulting in a smaller forward current. When the SiC trench MOSFETs operate under forward voltage, the p-n diode near the gate region is turned to the on state, and current conduction through the MCD and SBD is suppressed.

**Figure 4:**
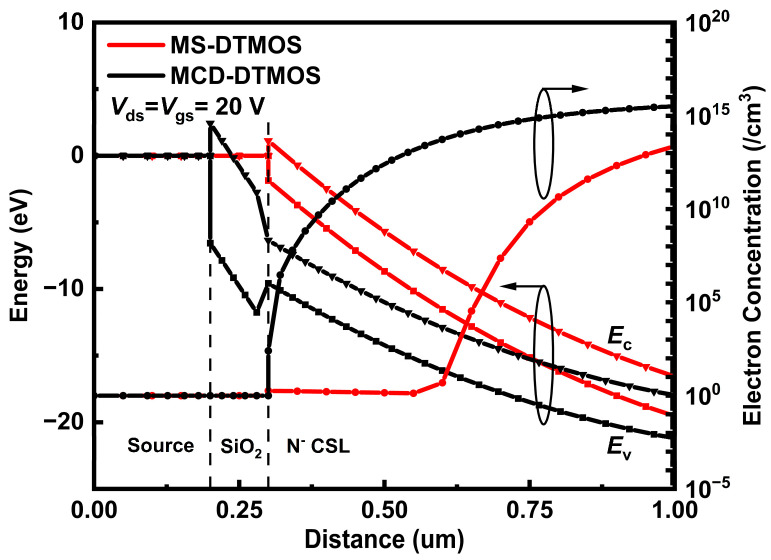
Energy band and electron concentration distribution at x = 0.2 μm of MS-DTMOS and MCD-DTMOS extracted from the source to drain.

Figure 4 shows the energy band diagrams extracted from source metal to N^−^ CSL in the MS-DTMOS and MCD-DTMOS biased at *V*_gs_ = *V*_ds_ = 20 V. For the MCD-DTMOS device, source metal–source oxide-N^−^ CSL achieve a metal–insulator–semiconductor (MIS) structure. For the MS-DTMOS device, the N^−^ CSL makes direct contact with the source metal and forms the Schottky barrier so that the conduction band is raised above the fermi energy level. Source oxide here can pull down the conduction band energy to below the fermi energy level so that the N^−^ CSL-side conduction band energy in MCD-DTMOS is lower than that in MS-DTMOS devices. So, the depleted region area of MS-DTMOS is larger than that of MCD-DTMOS. When the devices are output mode, the current conduction path in MS-DTMOS will be limited compared to MCD-DTMOS so that the forward output current in MS-DTMOS is slightly lower than that of MCD-DTMOS.

### 3.2. Breakdown Characteristics and Electric Field Distribution

**Figure 5:**
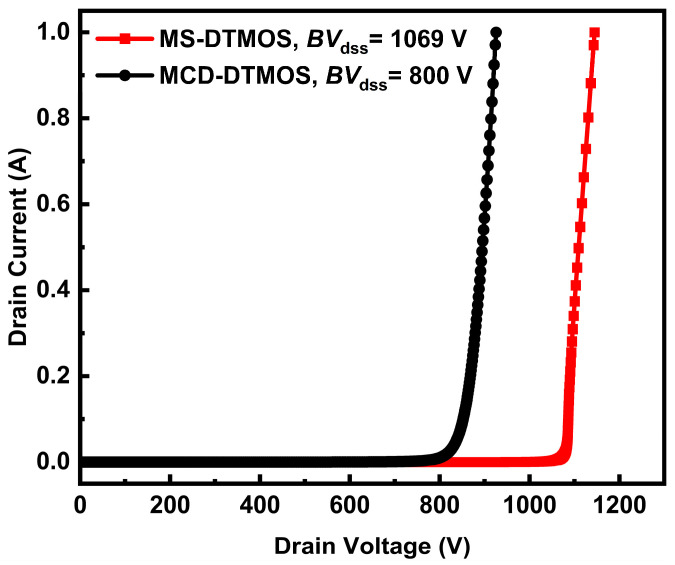
Breakdown characteristics of MS-DTMOS and MCD-DTMOS.

Figure 5 shows the off-state breakdown characteristics of the MS-DTMOS and the MCD-DTMOS. The breakdown voltage for MS-DTMOS is 1069 V, and for MCD-DTMOS, it is 800 V, which is an increase of 269 V.

**Figure 6:**
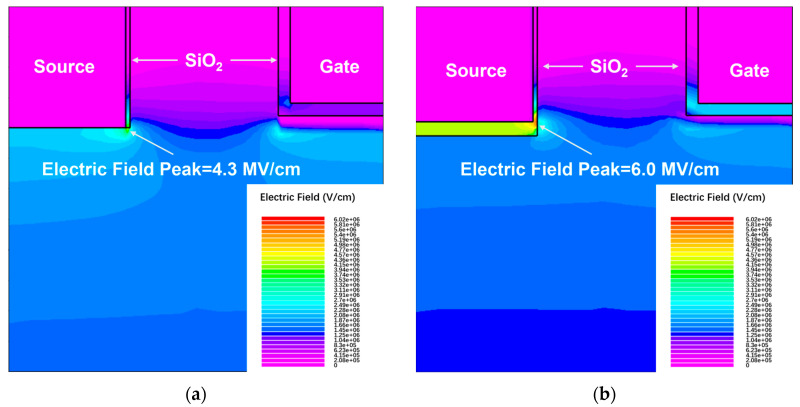
Electric field distribution of (**a**) MS-DTMOS at *V*_ds_ = 1069 V and (**b**) MCD-DTMOS at *V*_ds_ = 800 V.

To analyze the breakdown voltage characteristics, the electric field distributions at the off state, *V*_ds_ = *BV* is displayed in Figure 6. The improved breakdown characteristics stem from the optimized electric field distribution, where the Schottky contact in MS-DTMOS mitigates field crowding at the source trench bottom, reducing peak field intensity from 6.0 MV/cm of the conventional MCD-DTMOS to 4.3 MV/cm of the MS-DTMOS. For MCD-DTMOS, the electric field concentrates on the source oxide sidewall, which leads the device to break down earlier. In the source–N^−^ CSL Schottky junction, the electric field is governed by the depletion region modulated by the doping concentration and applied voltage, resulting in a smooth, laterally homogeneous distribution. Conversely, in the source–SiO_2_-N^−^ CSL MIS structure, the presence of the SiO_2_ layer introduces dielectric mismatch (ε_r,ox_ < ε_r,sic_), leading to localized field enhancement at SiO_2_ edges. Additionally, geometric fringe effects further distort the field in MIS structures. Consequently, MS-DTMOS achieves more predictable breakdown behavior, while MIS requires careful optimization of oxide quality and interface passivation to mitigate field non-uniformity and premature breakdown risks.

### 3.3. Parasitic Capacitance and Transient Characteristics

**Figure 7:**
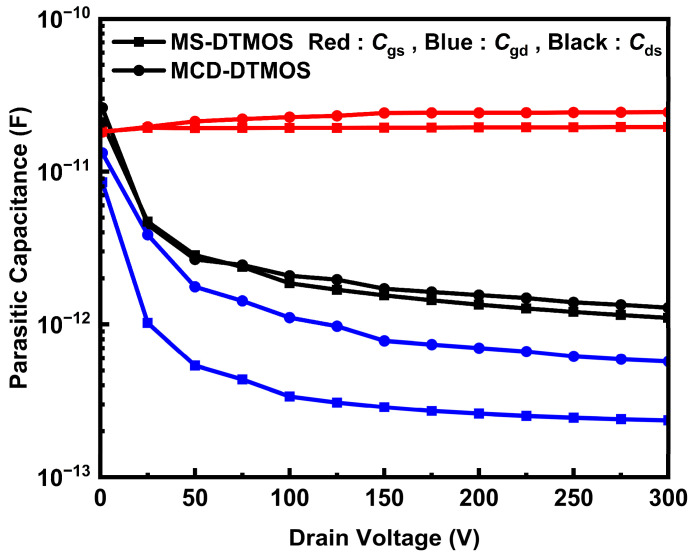
Parasitic capacitance characteristics biased at *V*_gs_ = 0 V of MS-DTMOS and MCD-DTMOS.

The parasitic capacitance characteristics of MS-DTMOS and MCD-DTMOS biased at 1 MHz, *V*_gs_ = 0 V, are exhibited in Figure 7. The drain-to-gate capacitance of MS-DTMOS is much lower due to the removal of the source button oxide layer [26]. Improvement in capacitance would result in better dynamic performance, indicating a better switching characteristic.

**Figure 8:**
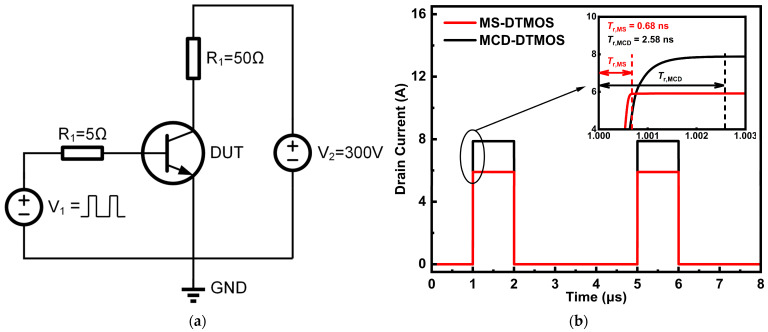
Turn-on switching test: (**a**) circuit and (**b**) switching characteristics of MS-DTMOS and MCD-DTMOS.

Figure 8 shows the turn-on switching test circuit and the switching characteristics of MS-DTMOS and MCD-DTMOS. The gate drive potential of DUT is a pulse rectangle wave with a 1 µs 15 V on-state signal and 3 µs −2 V signal off-state periodic operation. The rise time (*T*_r_) of MS-DTMOS is 0.68 ns compared with the 2.58 ns of MCD-DTMOS due to the lower parasitic capacitance of MS-DTMOS. Therefore, MS-DTMOS appears to have a faster response speed than MCD-DTMOS, which shows great potential to reduce energy loss.

**Figure 9:**
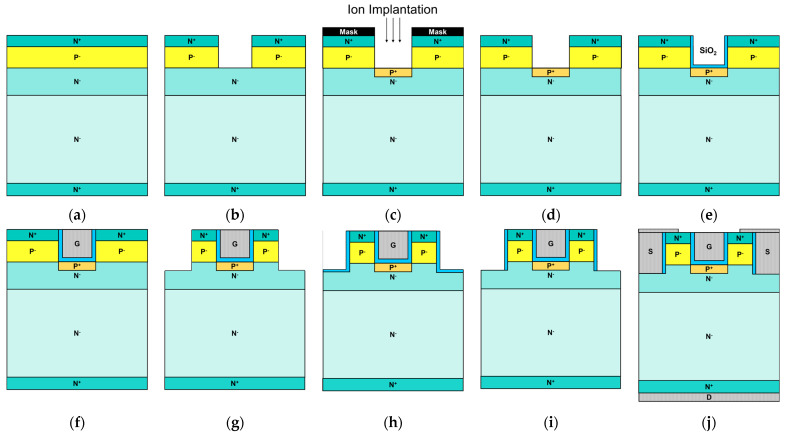
Proposed fabrication procedure steps of MS-DTMOS. (**a**) Epitaxial layer grown on 4H-SiC substrate. (**b**) Form trench by ICP-RIE. (**c**) Form p+ shielding region by implantation. (**d**) Mask removed by BOE wet etch. (**e**) Form gate oxide by thermal oxidation and ICP-RIE. (**f**) Form gate electrode by LPCVD. (**g**) Form trench by ICP-RIE. (**h**) Form source oxide by thermal oxidation. (**i**) Remove bottom oxide of source by ICP-RIE. (**j**) Form source and drain electrode by LPCVD.

Figure 9 illustrates a feasible fabrication procedure of the proposed MS-DTMOS. Here, epitaxial layers comprising a 9 µm N^−^ drift layer with an n-doped concentration of 7 × 10^15^ cm^−3^, a 1.4 µm N^−^ CSL layer with an n-doped concentration of 1 × 10^16^ cm^−3^, a 0.5 µm P^−^ base layer with a p-doped concentration of 3 × 10^16^ cm^−3^, and a 0.1 µm N^+^ layer with an n-doped concentration of 1 × 10^19^ cm^−3^ were grown on N^+^ 4H-SiC substrate with a doping concentration of 1 × 10^18^ cm^−3^, as shown in Figure 9a. Figure 9b displays that the gate trench region is created through inductively coupled plasma reactive ion etching (ICP-RIE). The P^+^ shielding layer is implanted using aluminum ions with a mask, as shown in Figure 9c. After the hard mask is removed, as shown in Figure 9d, the SiO_2_ layer is grown through low-pressure chemical vapor deposition (LPCVD) and region-selective etching as shown in Figure 9e. Using LPCVD, a p-type polysilicon layer is first deposited and subsequently etched back to create the lower polysilicon layer within the trench. Following this, n-type polysilicon is deposited to fill the trench and then etched back to achieve the configuration depicted in Figure 9f. With a similar procedure, the formation of the source trench region is completed, as displayed in Figure 9g, without the procedure of P+ implantation. Then, the SiO_2_ layer is grown through low-pressure chemical vapor deposition (LPCVD), as shown in Figure 9h, and region-selective etching, as shown in Figure 9i. Finally, the source and drain contact are formed after the isolation of the gate and source, as shown in Figure 9j. The described fabrication process outlines one potential implementation approach. However, substantial experimental verification and further development efforts remain necessary to realize the proposed device in practice.

Prior studies [27,28,29,30] have investigated various SiC MOSFET configurations with respect to their *R*_on_, BV, and switching performance characteristics. As summarized in Table 2, the proposed MS-DTMOS structure demonstrates superior performance, particularly in achieving the shortest *T*_r_ among all compared device structures, indicating its potential for significantly reduced switching energy losses

## 4. Conclusions

In this article, a novel 4H-SiC DTMOS with Integrated Schottky barrier and MOS-channel diodes is proposed and investigated using ATLAS TCAD simulations. The proposed MS-DTMOS achieves a breakdown voltage of 1069 V, which is 269 V higher than that of the MCD-DTMOS. The 2D electric field distributions profile shows that a mitigation of the electric field peak is observed, contributing to a higher breakdown voltage. Furthermore, due to the removal of a source oxide layer in the MS-DTMOS structure, the parasitic capacitance is reduced. Hence, the turn-on time of MS-DTMOS is 0.68 ns compared to the 2.68 ns of MCD-DTMOS, revealing the potential for lower switching energy loss. In summary, the MS-DTMOS architecture significantly enhances breakdown voltage and switching speed while maintaining acceptable forward conduction performance, offering a promising solution for high-frequency power converters. This design is particularly suited for electric vehicle inverters and renewable energy systems, where high efficiency and compact integration are critical.

## Figures and Tables

**Table 1:** Device parameters for simulations.

Parameters	Values
N^−^ Drift layer thickness (*T*_Drift_)	9 µm
N^−^ Drift layer concentration (*N*_Drift_)	7 × 10^15^/cm^3^
N^−^ CSL layer thickness (*T*_CSL_)	1.4 µm
N^−^ CSL Concentration (*N*_CSL_)	1 × 10^16^/cm^3^
Trench size	1.0 × 1.0 µm
Source length (*L*_source_)	1.0 µm
P^−^ base layer length (*L*_Base_)	0.63 µm
P^−^ base layer thickness (*T*_ch_)	0.5 µm
P^−^ base layer concentration (*N*_base_)	3 × 10^16^/cm^3^
N^+^/P^+^ concentration (*N*_N+_)	1 × 10^19^/cm^3^
N^+^ layer thickness (*T*_N+_)	0.1 µm
N^+^ Sub layer concentration (*N*_Sub_)	1 × 10^18^/cm^3^
N^+^ Sub layer thickness (*T*_Sub_)	1 µm

**Table 2:** Summary of the performance parameters of this work compared with previous work.

Parameters	Previous Work [27,28,29,30]				This Work
	[27]	[28]	[29]	[30]	
*R*_on_. _sp_ (mΩ·mm)	1.98	2.31	5.1	1.28	3.2
BV (V)	1200	1290	606	1560	1069
*T*_r_ (ns)	11	16.3	2.6	33.4	0.68

## Data Availability

Data are contained within the article.
